# Dramatic response and acquired resistance to savolitinib in advanced intrahepatic cholangiocarcinoma with MET amplification: a case report and literature review

**DOI:** 10.3389/fonc.2023.1254026

**Published:** 2023-11-02

**Authors:** Kexun Zhou, Yingping Liu, Hong Zhu

**Affiliations:** ^1^ Department of Medical Oncology, Cancer Center, West China Hospital, Sichuan University, Chengdu, China; ^2^ The Second Clinical Medical College of Lanzhou University, Lanzhou University, Lanzhou, China

**Keywords:** biomarker-guided treatment, savolitinib, intrahepatic cholangiocarcinoma, MET amplification, resistance

## Abstract

**Background:**

Cholangiocarcinoma (CCA) is an aggressive disease with limited treatment options. Despite substantial efforts to explore better regimens, gemcitabine-based chemotherapy has been the standard first-line treatment for decades. With the growing field of precision medicine, biomarker-guided treatments are gaining popularity. MET alteration is a frequent occurrence in various cancer types, making it a promising target.

**Case presentation:**

A 53-year-old man visited our hospital with a complaint of upper abdominal pain. Advanced CCA was diagnosed based on the biopsy of the metastatic lymph nodes and immunohistochemistry. Next-generation sequencing revealed MET amplification. As the patient was intolerant to traditional chemotherapy, savolitinib (a c-MET inhibitor) was administered. Partial response was achieved, and the treatment was well tolerated. After 1 year, the patient developed progressive disease, to which the emergence of epidermal growth factor receptor amplification may have contributed.

**Conclusion:**

Our study verified the therapeutic value of a c-MET inhibitor in advanced CCA-harboring MET amplification and provides an alternative strategy for patients who are intolerant to chemotherapy.

## 1 Introduction

Cholangiocarcinoma (CCA), including intrahepatic CCA (iCCA), perihilar CCA (pCCA), and distal CCA (dCCA), is characterized as a low-incidence malignancy, but has a poor prognosis, with a 5-year overall survival (OS) rate of less than 20%. The incidence of iCCA is gradually increasing, and most cases are diagnosed at an advanced stage (1).

In recent years, with the development of comprehensive genomic profiling, biomarker-guided treatment for CCA has increased. Genetic alterations, including fibroblast growth factor receptor 2 (FGFR2) and isocitrate dehydrogenase (IDH), are noteworthy in CCA. Based on the results of clinical trials, ivosidenib has been approved for CCA harboring an IDH-1 mutation, whereas pemigatinib is recommended for CCA with FGFR2 fusions or rearrangements. Furthermore, a neurotrophic tropomyosin-receptor kinase (NTRK) inhibitor and a combination of BRAF and MEK inhibitors also yielded a survival benefit in advanced CCA (2).

MET is a proto-oncogene associated with the progression of various cancers. Significant efforts have been made to develop inhibitors targeting MET, and savolitinib is a novel example. Based on the results of a phase III trial in China, savolitinib was approved for metastatic non-small-cell lung cancer (NSCLC) with MET exon 14-skipping alterations (3). For CCA, high-level c-Met amplification is rare, with an incidence of less than 2% (4). Despite this, a previous study suggested that high c-MET expression in CCA is associated with inferior outcomes, and could be an independent risk factor for OS and disease-free survival (DFS) (5). These results contradicted those of Pu et al. (6).

However, evidence regarding the efficacy of savolitinib in the treatment of advanced CCA is lacking. Here, we report a case of advanced iCCA harboring MET amplification that was treated with savolitinib and achieved a partial response (PR) with manageable adverse events (AEs). Epidermal growth factor receptor (EGFR) amplification during disease progression may contribute to the development of acquired resistance to savolitinib.

## 2 Case presentation

**Figure 1:**
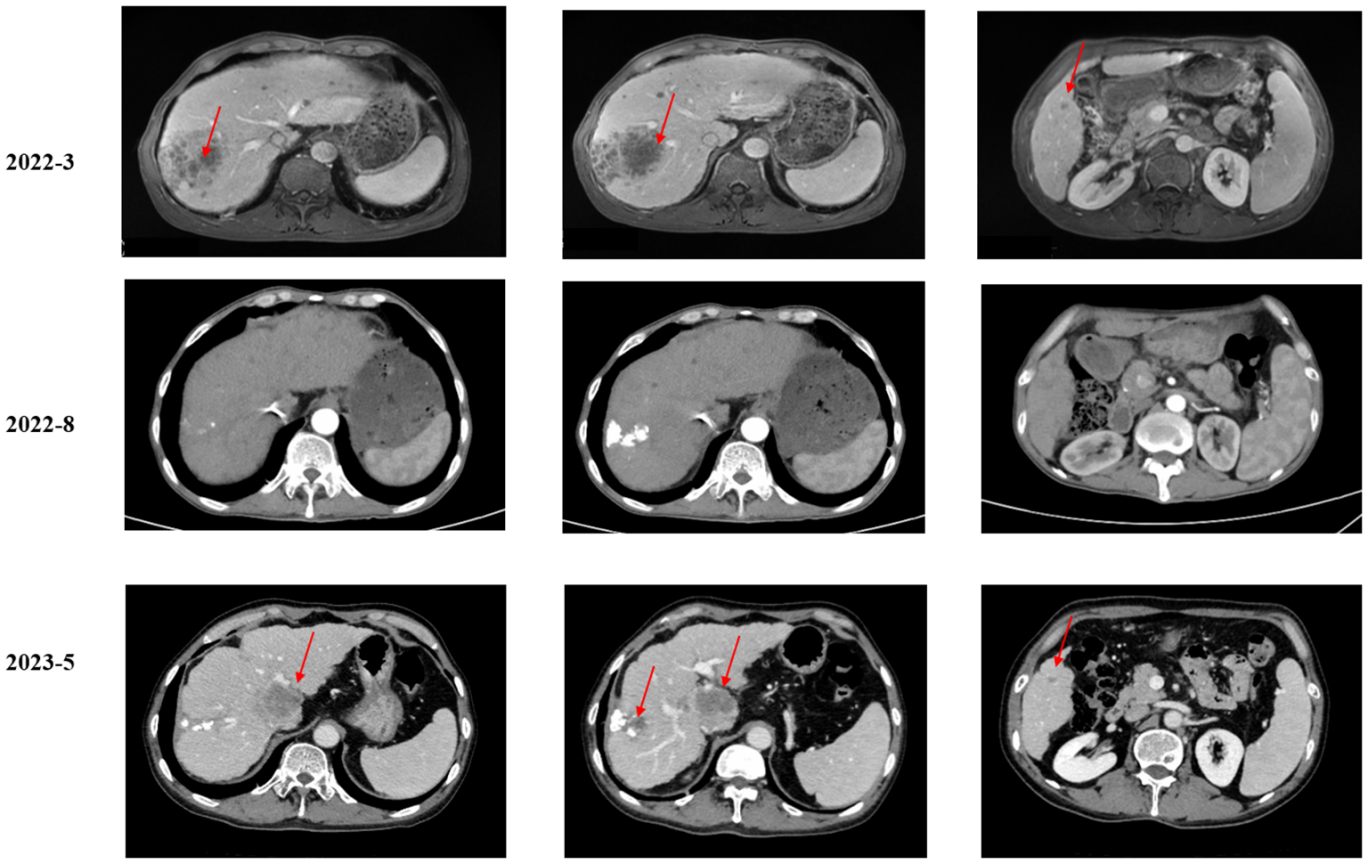
Imaging examination of the liver mass.

A 53-year-old man presented to a local hospital with the complaint of upper abdominal pain. The patient had no significant medical history. However, he had a family history of cancer, as both his brothers had died of liver cancer. Imaging revealed multiple masses in the liver. The patient visited our institution for confirmation of the diagnosis. Magnetic resonance imaging (MRI) of the abdomen confirmed a mass in the right lobe of the liver with multiple intrahepatic nodules and lymph node metastases. Computed tomography (CT) of the chest revealed multiple nodules and lymph nodes in the left supraclavicular fossa and mediastinum, suspicious of metastatic disease (**Figure 1**). Biopsy of lymph nodes was performed. Immunohistochemistry (IHC) showed CK7 (+), CK20 (+), HepPar-1 (−), SATB2 (−), CDX2 (−), TTF-1(8G7G3/1) (−), Pax-8 (−), NKX3.1 (−), MLH1 (+), MSH2 (+), MSH6 (+), PMS2 (+), and HER2 (0). Therefore, the patient was diagnosed with advanced iCCA. Next-generation sequencing (NGS) results suggested that the patient harbored MET amplification and that the tumor mutation burden (TMB) was high (TMB-H, 11.52 Muts/Mb) (**Supplementary Table 1**).

**Figure 2:**
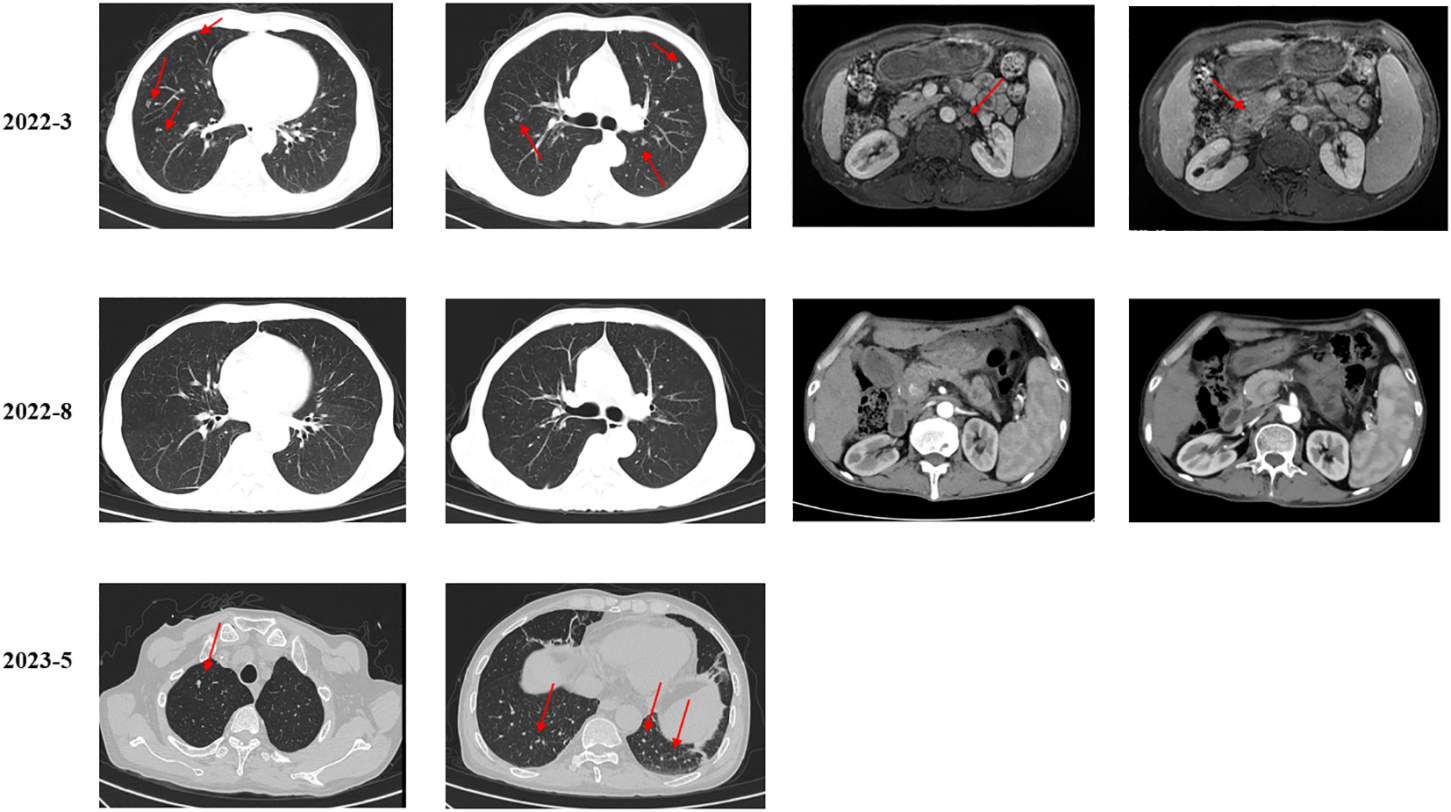
Imaging examination of the lung metastasis and lymph nodes.

**Figure 3:**
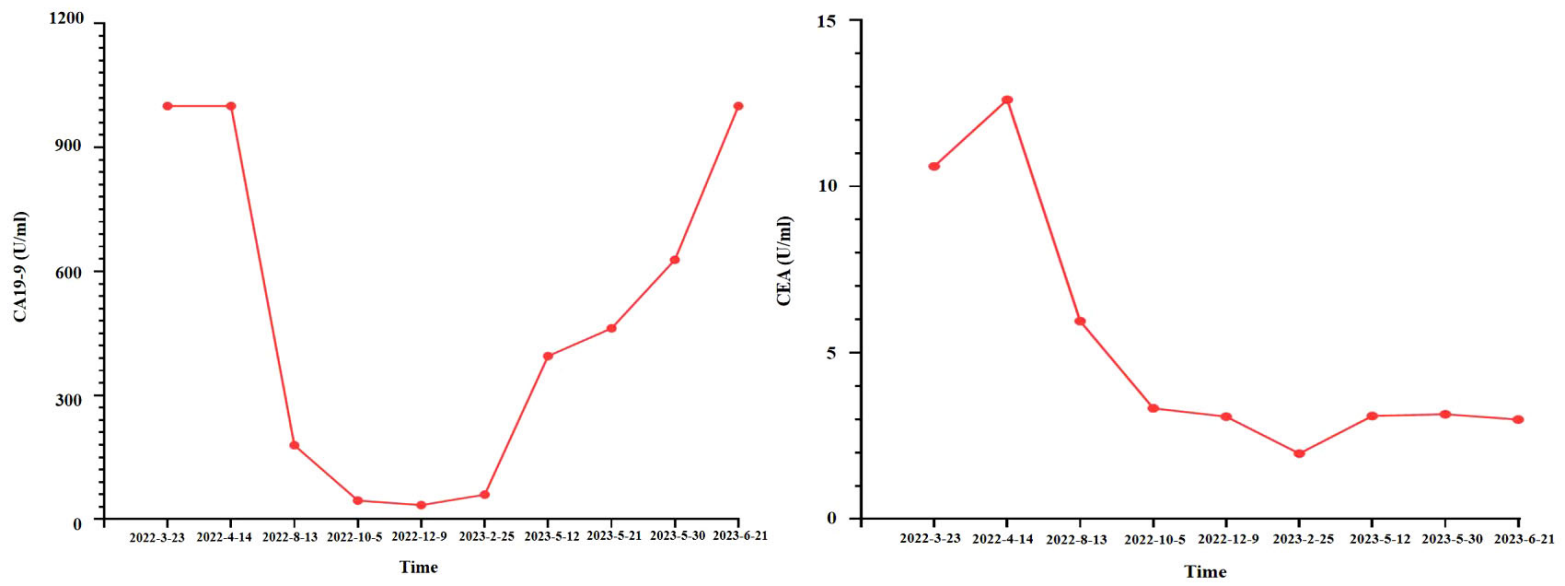
The trend of serum CA19-9 and CEA.

As suggested by a multidisciplinary team (MDT), transarterial chemoembolization (TACE) was performed on 29 March 2022. Combined therapy with gemcitabine (1,000 mg/m^2^ on days 1 and 8, q3w) and cisplatin (25 mg/m^2^ on day 1, q3w) was administered in April 2022. After three cycles, the local image monitor suggested stable disease. However, the patient complained of poor treatment tolerance, as grade 2 nausea, vomiting, and grade 3 decreased platelet occurred. When the dosage was reduced by 25%, the situation was not improved. The patient still suffered from grade 3 myelosuppression. Thus, treatment was switched to nab-paclitaxel (125 mg/m^2^ on days 1, 8, and 15, q4w). Unfortunately, the patient experienced grade ≥3 myelosuppression after one cycle. Treatment with cytotoxic chemotherapy was not considered because of intolerable adverse effects. Based on the NGS results, MET inhibitors and immunotherapy were considered as alternative treatments. After a discussion with the patient and his family, they preferred savolitinib. Treatment with savolitinib (600 mg/day) was initiated in June 2022. After 2 months of treatment, PR was achieved (according to RECIST version 1.1) as the liver mass dramatically decreased (**Figure 1**). Meanwhile, the lymph nodes in the abdomen and lung masses also disappeared (**Figure 2**). Serum carbohydrate antigen (CA) 19-9 and carcinoembryonic antigen (CEA) levels also remarkably decreased (**Figure 3**). A follow-up CT scan indicated continuous PR. Savolitinib was well-tolerated and no grade ≥3 AEs occurred.

**Figure 4:**
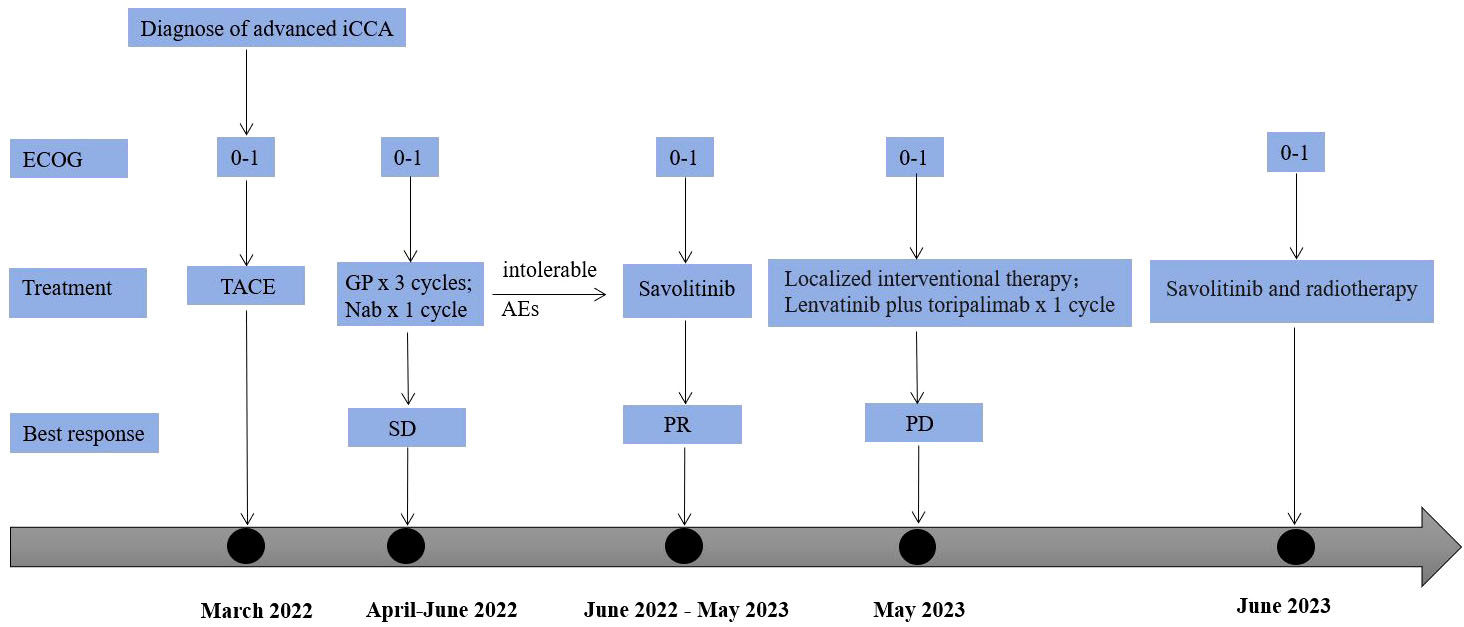
A timeline of this clinical case. ECOG, Eastern Cooperative Oncology Group iCCA; intrahepatic cholangiocarcinoma; TACE, transarterial chemoembolization; GP, gemcitabine plus cisplatin; Nab, nab-paclitaxel; SD, stable disease; AEs, adverse events; PR, partial response; PD, progression disease.

However, in May 2023, new nodules were identified in the liver and lungs, implying the experience of disease progressive disease (PD) (according to RECIST version 1.1) (**Figure 1**). As suggested by the MDT, localized interventional therapy was recommended because the performance status of the patient was good (Eastern Cooperative Oncology Group [ECOG] 0–1). Immunotherapy was considered as a subsequent treatment because the patient harbored high TMB. Additionally, to understand the mechanism of acquired resistance to savolitinib, NGS was performed. The NGS results indicated that the patient still harbored MET amplification. EGFR amplification was also detected (**Supplementary Table 2**). After recovering from the effect of localized therapy, the patient was administered lenvatinib plus toripalimab. However, the liver masses were not under control after one cycle. Based on the latest NGS results, cetuximab was recommended. However, the patient and his family could not afford the treatment and rejected this recommendation. Since savolitinib is widely used in treating lung cancer, we consulted a thoracic oncologist for advise on further treatment. Based on their clinical practice experience, savolitinib could be continued and localized radiotherapy was recommended. The patient and his family were fully informed of the current alternative treatment and prognosis, and agreed to receive savolitinib and radiotherapy. Savolitinib was restarted in June 2023, combined with radiotherapy. The timeline of this clinical case is summarized in **Figure 4**.

## 3 Discussion

To the best of our knowledge, this is the first case report describing a significant clinical response to savolitinib in a patient with MET-amplified advanced iCCA who was intolerant to traditional chemotherapy. The efficacy of savolitinib was maintained for nearly 1 year.

Gemcitabine-based chemotherapy is the standard first-line treatment for advanced CCA, and a minority of patients harboring genetic alterations are eligible for treatment with tyrosine kinase inhibitors (TKIs). Although high level c-Met amplification is rare in CCA, its activation could promote cancer development (4, 7). Thus, in the era of targeted therapy, hope is on the inhibition of the MET pathway. However, the efficacy of MET inhibitors in treating CCA remains controversial. Tivantinib is a small-molecule inhibitor of c-MET kinase. In a preclinical study, Wei et al. found that tivantinib inhibited the growth of CCA cell lines, and those with high c-MET expression were more sensitive to tivantinib (8). In a phase I study, tivantinib combined with gemcitabine was safe and tolerable in patients with advanced solid tumors, including CCA (9). However, there have been no further phase III trials to verify this result. Cabozantinib is a receptor TKI with activity against MET, VEGFR2, FLT3, c-KIT, and RET. In a phase II trial, cabozantinib yielded limited success in patients with CCA, but further analysis indicated that one patient with a MET-high tumor had a prolonged benefit from treatment (10). In addition, when combined with gemcitabine and cisplatin, merestinib, another MET inhibitor, did not improve the outcomes (11). In contrast, two case reports have shown that patients with advanced CCA with MET alterations showed dramatic response to capmatinib, a selective inhibitor of MET receptor. The case report published by Turpin et al. described a PR in a patient with iCCA and CAPZA-2-MET fusion who experienced progression after second-line treatment (12). Lefler et al. also reported that PR was achieved in a patient with advanced iCCA harboring high-level MET amplification (13). These results suggest that our current understanding of MET inhibitors in CCA is inadequate.

Savolitinib has potent MET inhibitory properties. In addition to NSCLC, its efficacy and safety have been evaluated in other cancer types. In the SAVOIR trial, savolitinib achieved longer PFS and OS in patients with papillary renal cell carcinoma than sunitinib (14). A few previous studies have indicated that savolitinib achieved optimal results in patients with MET-driven advanced gastric cancer (GC). Ye et al. reported a middle-aged man with advanced GC who was intolerant to chemotherapy and immunotherapy. NGS identified MET amplification and rearrangement, and savolitinib was administered. The patient achieved PR with an improved general condition. They also reported the efficacy of savolitinib as a third-line treatment in young women with advanced GC who harbored MET amplification. The CT scan taken 2 months later showed a PR, and at the time of reporting, the patient was alive and remained progression-free for 3 months (15). The results were consistent with that of the case reported by He et al., where a 35-year-old male with advanced GC and MET amplification received savolitinib as first-line treatment and achieved PR (16). Notably, the efficacy of savolitinib in our study was maintained for nearly 1 year, which is much longer than that reported for capmatinib. Accordingly, it is reasonable to expect savolitinib to show a more optimal outcome in patients with iCCA who harbor MET amplification. However, we must clarify that, since the chemotherapies were stopped due to toxicities, rather than disease progression, their extended benefit could not be excluded from the patient’s duration of response. Further studies are needed to validate our findings.

Notably, many patients succumb to PD after TKI treatment, motivating the efforts towards the development of subsequent generation TKIs. Frigault et al. explored the mechanisms of resistance to savolitinib in advanced GC. Using circulating tumor DNA (ctDNA) sequencing, the authors found that when patients experienced PD after treatment with savolitinib, MET D1228V/N/H and Y1230C mutations were identified, as well as a high copy number of MET amplification. This may contribute to resistance to savolitinib (17). In addition, Han et al. suggested that FGFR1, EGFR, and KRAS amplification emergence was likely responsible for the resistance to savolitinib in a patient with pulmonary sarcomatoid carcinoma (18). A preclinical study proposed that switching to EGFR dependence or a requirement for PIM signaling is associated with the resistance to savolitinib mechanisms in NSCLC (19). Our study results are consistent with these findings, as EGFR amplification was identified at the time of PD. Notably, the patient in the current study preferred to restart savolitinib treatment combined with radiotherapy after PD. There is no evidence on whether previously treated patients can benefit from savolitinib. Therefore, the patient will be closely followed.

Overall, this case validates the efficacy of savolitinib for advanced iCCA with MET amplification, providing an alternative treatment for this patient population. Further studies are required to confirm these findings.

## Data availability statement

The original contributions presented in the study are included in the article/**Supplementary Material**. Further inquiries can be directed to the corresponding author.

## Ethics statement

The studies involving humans were approved by the Institutional Ethics Review Board of West China Hospital, Sichuan University. The studies were conducted in accordance with the local legislation and institutional requirements. The participants provided their written informed consent to participate in this study. Written informed consent was obtained from the individual(s) for the publication of any potentially identifiable images or data included in this article.

## Author contributions

KZ: Writing – original draft. YL: Writing – original draft. HZ: Supervision, Visualization, Writing – review & editing.
