# Supplementary material for: Dramatic response and acquired resistance to savolitinib in advanced intrahepatic cholangiocarcinoma with MET amplification: a case report and literature review
Source: Front Oncol. 2023 Nov 2;13:1254026. doi: 10.3389/fonc.2023.1254026 (PMC10652553; doi:10.3389/fonc.2023.1254026)
Supplement: Supplementary file 2 [file Table_2.docx]

**Table 2 The results of NGS after disease progression**

| **Genes** | **Alterations** | **Results (variant allele frequencies)** |
| --- | --- | --- |
| EGFR | Exon 18 | - |
|  | Exon 19 | - |
|  | Exon 20 (including T790M) | - |
|  | Exon 21 | - |
|  | Amplification | + (12.8) |
| ERBB2 (HER2) | Amplification | - |
|  | Mutation | - |
| ALK | Rearrangement | - |
| ROS1 | Rearrangement | - |
| MET | Amplification | + (5.0) |
| RET | Rearrangement | - |
| BRAF | V600E | - |
| KIT | Exon 9 | - |
|  | Exon 11 | - |
|  | Exon 13 | - |
|  | Exon 17 | - |
| PDGFRA | Exon 12 | - |
|  | Exon 18 | - |
| BRCA1 | Mutation | - |
| BRCA2 | Mutation | - |
| KRAS | Mutation | - |
| NRAS | Mutation | - |
| PIK3CA | Mutation | - |
| FGFR2 | Rearrangement | - |
|  | Mutation | - |
| FGFR3 | Rearrangement | - |
|  | Mutation | - |
| NTRK1 | Rearrangement | - |
| NTRK2 | Rearrangement | - |
| NTRK3 | Rearrangement | - |
| IDH1 | Mutation | - |

NGS, Next-generation Sequencing
